# Association between Dental Caries and Influenza Infection in Children: A Japanese Nationwide Population-Based Study

**DOI:** 10.3390/children8090780

**Published:** 2021-09-06

**Authors:** Naomi Matsumoto, Tomoka Kadowaki, Hirokazu Tsukahara, Takashi Yorifuji

**Affiliations:** 1 Department of Epidemiology, Graduate School of Medicine, Dentistry and Pharmaceutical Sciences, Okayama University, Okayama 700-8558, Japan; tomokaden232@gmail.com (T.K.); yorichan@md.okayama-u.ac.jp (T.Y.); 2 Department of Pediatrics, Graduate School of Medicine, Dentistry and Pharmaceutical Sciences, Okayama University, Okayama 700-8558, Japan; tsukah-h@cc.okayama-u.ac.jp

**Keywords:** dental caries, influenza, birth cohort, oral health

## Abstract

Dental caries is the most common chronic childhood disease. Recent studies have suggested that dental caries harbor respiratory infections in adults. We investigated the association between dental caries and influenza in children. In this study, 42,812 children aged 2.5 years, 38,540 children aged 5.5 years, and 34,124 children aged 10 years were included in the analysis from the Longitudinal Survey of Newborns in the 21st Century in Japan, which targeted all children born during a certain period in 2001. We used information on dental caries treated at hospitals and clinics in the past year as exposure and influenza as outcome during the observation periods (1.5–2.5, 4.5–5.5, and 9–10 years of age). We performed a log-binomial regression analysis, adjusting for potential confounders, and stratified analysis according to previous dental caries status. The presence of dental caries increased the incidence of influenza in all three target ages compared with the absence of dental caries. The incidence of influenza increased with the presence of current dental caries, regardless of the presence of past dental caries. These associations were observed irrespective of household income. Early detection and treatment of dental caries may reduce the risk of influenza in children.

## 1. Introduction

Oral health is fundamental to overall health, well-being, and quality of life [1]. According to the 2019 Global Burden of Diseases study, caries of the permanent teeth was the most prevalent disease (affecting 2.0 billion people worldwide) and caries of the deciduous teeth was the 18th most prevalent disease (affecting 530 million children worldwide) in all ages combined [2]. Dental caries is associated with negative child and family experiences and lower quality of life for the child and their caregivers [3,4]. It has recently been reported that the oral cavity may harbor manifestations of systemic diseases such as cardiovascular disease and metabolic syndrome [5,6].

There is fair evidence of an association of respiratory infection, especially pneumonia, with oral health, particularly in elderly adults [7,8]. Even in young adults, dental caries increases the risk of lower respiratory tract infections [9]. In the field of pediatrics, however, few studies have focused on the association of respiratory infection with dental caries and there have been even fewer studies on the association of dental caries with increased risk of influenza infection.

In the present study, therefore, we investigated the association between dental caries and influenza using a large nationwide birth cohort in Japan.

## 2. Materials and Methods

### 2.1. Participants

The Japanese Ministry of Health, Labor and Welfare has been conducting “The Longitudinal Survey of Babies in the 21st Century” since 2001 to establish political strategies that can be taken to address the declining birthrate in Japan [10,11,12,13,14]. This survey included all children born in Japan between 10 and 17 January or 10 and 17 July in 2001. Baseline questionnaires were mailed to 53,575 families when eligible children were 6 months old. Of those families, 47,015 answered the baseline questionnaires (88% response rate) that provided perinatal information as well as information on biological and socioeconomic status including parental academic attainment and parental smoking status. Birth record data from the vital statistics system of Japan are also linked with each targeted child.

**Figure 1:**
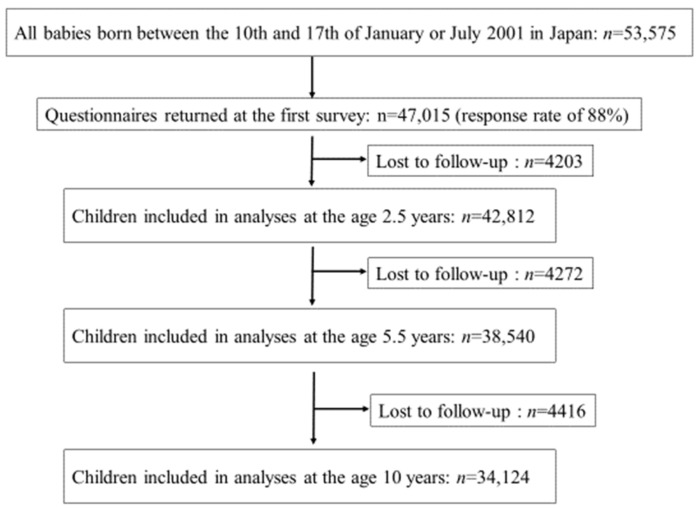
Flowchart of participants.

The respondents were annually mailed follow-up questionnaires to investigate children’s medical conditions and behaviors at their birth months. Of them, we included children whose families answered the baseline questionnaire and the follow-up questionnaire at each targeted age (at 2.5, 5.5, and 10 years of age) to investigate the role of dental caries in influenza infection at various ages. The paper questionnaire was completed by the children’s guardians. From this nationwide population-based study, we included 42,812 children at the age of 2.5 years, 38,540 children at the age of 5.5 years, and 34,124 children at the age of 10 years in the analysis (Figure 1).

### 2.2. Dental Caries

In the second to tenth surveys, the questionnaire included a question about whether the targeted child experienced medical consultation at an outpatient clinic for various common diseases such as dental caries. Respondents marked up the assigned number of dental caries in the questionnaire when their children had visited the dentist for treating dental caries during the past 12 months. To investigate the associations between dental caries and influenza for a wide age distribution, we targeted three observational ages (ages of 2.5, 5.5, and 10 years). Then, we could consequently use information on dental caries that have been examined at hospitals and clinics for the periods during 1.5 to 2.5, 4.5 to 5.5, and 9 to 10 years of age. Information on the number, severity and treatment methods of oral caries was not available from the questionnaires. Since the Japanese national health insurance system covers dental care and children usually have an annual dental examination in daycare centers, kindergartens and primary schools [15], information on dental caries status from the annual questionnaires should be valid.

### 2.3. Influenza

In a similar way, we used information on child’s visit to the doctor for treating influenza infection across the three observation periods as the outcome of interest. The amount of oseltamivir prescribed in Japan in the 2000s accounted for 80% of the amount prescribed worldwide, and Japanese patients with flu symptoms generally consult with a medical doctor to have an influenza antigen test and receive a prescription of anti-influenza medicine [16,17]. This unique custom in Japan indicates that the data from the annual self-reports about influenza infection are valid.

### 2.4. Statistical Analyses

We first compared baseline characteristics of children with and those without dental caries between the ages of 18 and 30 months. We then conducted a log-binomial regression analysis to investigate the association between dental caries status and children’s outpatient visits for influenza during the same observational periods, i.e., during the periods from 1.5 to 2.5, 4.5 to 5.5, and 9 to 10 years of age. We estimated crude risk ratios (RRs) and their 95% confidence intervals (CIs) and then estimated adjusted risk ratios (aRRs) with control for potential child confounders (Model 1) and potential child and parental confounders (Model 2).

We selected the following child and parental factors as potential confounding factors. Child factors included sex (dichotomous), singleton or multiple birth (dichotomous), term or preterm birth (<37 weeks of gestation; dichotomous), parity including the targeted child (1, 2, ≥3; categorical), family numbers without the targeted child (1, 2, 3–5, ≥6; categorical), infant feeding practices (exclusive breastfeeding, mixed breastfeeding, formula only; categorical), place of birth and residence (ward, city, town, or village; categorical), and daycare attendance at 1.5 years of age (dichotomous). Parental factors included maternal and paternal ages at delivery (<25, 25–29, 30–34, and ≥35 years; categorical), maternal and paternal educational attainment (university or higher, junior college, high school, junior high school, or other; categorical) and maternal smoking status (non-smoker, smoking <10 cigarettes per day, and smoking ≥10 cigarettes per day; categorical). We obtained information on birth weight, sex, parity, maternal, and paternal ages at delivery, and places of birth and residence from birth records. Information on parity, family numbers, infant feeding practices and maternal smoking status was obtained in the first survey, and information on maternal and paternal educational attainment and daycare attendance was obtained in the second survey. Information on family numbers was obtained from the corresponding survey (i.e., third survey for 2.5 years of age, sixth survey for 5.5 years of age, and tenth survey for 10 years of age). These potential confounders were selected on the basis of earlier studies or prior knowledge of the association between dental caries and influenza [18,19]. Cases with missing data were excluded and we conducted our analysis with complete cases only.

To further examine the impact of dental caries status on influenza infection during the same observational periods, we stratified the participants by past dental caries and repeated the analysis. To increase the proportion of children with past dental caries, we restricted the analysis to the observational period between 9 and 10 years of age and defined children with past dental caries as those who had caries examined at hospitals and clinics between 1.5 and 9 years of age.

Because low socio-economic status (SES) can increase the risk of both oral caries and influenza [18,19], SES can confound the association between dental caries and influenza infection. Although we adjusted for maternal and paternal educational attainment in the main analysis, we further conducted stratified analysis by house income in the sensitivity analysis. Because information on income was only obtained in the tenth survey (at the age of 10 years), we restricted the analysis to the observational period between 9 and 10 years of age. After calculating the house income by combining mother’s, father’s, and other annual house income, we stratified the participants by three categories of house income, i.e., low income (under 25%), middle income (25% to 75%) and high income (over 75%).

Statistical analyses were performed using Stata version 16 (StataCorp LLC, College Station, TX, USA). This study was conducted in accordance with the Declaration of Helsinki, and the protocol was approved by the Institutional Review Board at Okayama University Graduate School of Medicine, Dentistry, and Pharmaceutical Sciences (No. 1506-073).

## 3. Results

### 3.1. Participant Characteristics

Participants’ demographic characteristics according to dental caries status between the ages of 1.5 and 2.5 years are shown in Table 1. Compared to children without dental caries, children with dental caries tended to have older siblings and more family members, to have been fed with exclusive breastfeeding, to have gone to daycare at 1.5 years of age, to live in towns or villages, and to have parents with lower academic attainment and higher rates of maternal smoking.

There were 2 cases with missing information for family members, 324 cases with missing information for infant feeding practices, 97 cases with missing information for daycare attendance, 219 cases with missing information for maternal smoking, 1374 cases with missing information for maternal educational attainment, and 1790 cases with missing information for paternal educational attainment.

### 3.2. Dental Caries and Influenza

The RRs and aRRs from the log-binomial regression analysis are shown in Table 2. The presence of dental caries increased the incidence of influenza in all three targeted ages compared to the absence of dental caries even after adjustment for both child and parental factors. The aRRs were 1.15 (95% CI: 1.05–1.25) for 1.5–2.5 years of age, 1.06 (95% CI: 1.01–1.11) for 4.5–5.5 years of age, and 1.22 (95% CI: 1.17–1.28) for 9–10 years of age.

### 3.3. Subgroup Analysis by Past Dental Caries Status

The subgroup analysis of children aged 9 to 10 years according to the past status of the dental caries is shown in Table 3. Present dental caries increased the risk of influenza infection in children with no past dental caries compared to the reference group without both present and past dental caries (aRR 1.23; 95% CI: 1.08–1.41). In contrast, having past dental caries did not increase the risk of influenza infection in children without recent dental caries (aRR 1.01; 95% CI: 0.95–1.07). These results suggested the importance of the presence of dental caries during the corresponding period for influenza infection.

### 3.4. Sensitivity Analysis (Stratified by the Household Income Categories)

In the sensitivity analysis shown by Table 4, the presence of dental caries increased the incidence of influenza in all household income categories (aRR for low income group 1.34; 95% CI: 1.21–1.48, aRR for middle income group 1.19; 95% CI: 1.11–1.27, aRR for high income group 1.16; 95% CI 1.04–1.28).

## 4. Discussion

In the present study, we investigated whether the presence of dental caries was associated with the incidence of influenza. We found that the presence of dental caries increased the incidence of influenza compared with the absence of dental caries in all of the three targeted ages. Moreover, an increased risk was observed regardless of the presence of past dental caries or the category of SES status.

There is fair evidence of an association of pneumonia with dental decay in elderly people. For example, one study showed that the incidence of pneumonia increased by 1.2 times per one decayed tooth [7]. Moreover, a cohort study including young Finnish adults aged 20–27 years suggested that dental caries increased the risk of lower respiratory tract infections even among young adults [9]. In the field of pediatrics, there have been few studies that focused on the association of respiratory infection with dental caries, but the results of one study showed that dental caries experience was associated with reduced risk of upper respiratory infection in preschool children, results that are not consistent results for adults [20] and our results. Possible reasons for the discrepancy in results are differences in sample size, residual confounding, and targeted outcome of upper respiratory infection. Further studies targeting young children are needed. Our study investigated the association between dental caries and influenza in children using a large nationwide birth cohort in Japan.

One possible reason for why oral cavities increased the risk of influenza infection is the influence of poor oral health. HA (hemagglutinin) and NA (neuraminidase) on the surface of the influenza virus play an important role in viral entry and release [21,22], and TLP (trypsin-like proteases) produced by oral bacteria (e.g., Staphylococcus aureus, Pseudomonas aeruginosa, and pneumococcus) mediate influenza virus HA modification to activate infection [23,24]. Abe et al. showed that professional oral health care reduced activities of both NA and TLP in saliva, which led to reduced influenza risk among elderly persons [25]. Poor oral hygiene (one of the risk factors of oral caries), not oral caries itself, may increase the risk of influenza [26]. Our results that the absence of current dental caries, even in children who had previous dental caries, did not increase the incidence of influenza, support this hypothesis. Future studies regarding poor oral hygiene and risk of respiratory infections including influenza are also warranted.

The strength of the present study is its inclusion of participants from a large nationwide population-based study. In addition, we extensively adjusted for possible biological and socioeconomic confounders. Since both dental caries and influenza are associated with socioeconomic factors, adjustment for socioeconomic confounders is crucial.

However, our study also has some limitations. First, information on dental caries and influenza was obtained from parental reports, not from clinically confirmed methods. This could have resulted in misclassification. However, Japanese patients with flu symptoms generally consult with a medical doctor to have an influenza antigen test, and Japanese children normally have annual dental examinations in educational settings. Thus, the possible misclassification would be small and moving the effect estimates toward the null. Second, there was no information about history of periodontal disease. Periodontal disease could be responsible for the development of various systemic diseases and it can be a confounder [5,27]. Future studies that include information on periodontal disease history and/or oral hygiene itself should be performed. Third, since the variable of dental caries in our study is the presence or absence of child’s visit to the dentist for treating dental caries, it may reflect not only the occurrence of dental caries but also the health-seeking behavior of caregivers toward their children. We have addressed this issue by adjusting for socioeconomic factors but there remains the issue of further unmeasured confounding. Finally, we studied Japanese children, and the generalizability of our findings to other populations is unclear.

Dental caries is the most common chronic childhood disease. The Surgeon General’s Report on Oral Health 2000 made it clear that oral health is a part of overall health and well-being [28]. The present study suggested the importance of dental caries in the incidence of influenza infection and particularly acute infectious diseases in childhood. The findings reinforce the importance of oral health for children’s health.

## 5. Conclusions

In the present study, we demonstrated that the presence of current dental caries increased the incidence of influenza regardless of the presence of past dental caries. Early detection and treatment of dental caries may reduce the risk of influenza in children.

## Figures and Tables

**Table 1:** Demographic Characteristics of Eligible Children According to Caries Status (*N* = 42,812).

	Total	Caries (−) between the Ages of 1.5 and 2.5 Years	Caries (+) between the Ages of 1.5 and 2.5 Years
	(*N* = 42,812)	(*N* = 39,776)	(*N* = 3036)
Characteristics of children			
Gender, *n* (%) *			
Boys	22,216 (51.89)	20,594 (51.77)	1622 (53.43)
Girls	20,596 (48.11)	19,182 (48.23)	1414 (46.57)
Singleton or multiple birth, *n* (%) *			
Singleton birth, *n* (%)	41,955 (98.00)	38,949 (97.92)	3006 (99.01)
Multiple birth, *n* (%)	857 (2.00)	827 (2.08)	30 (0.99)
Term or preterm birth, *n* (%) *			
Term birth, *n* (%)	40,698 (95.06)	37,773 (94.96)	2925 (96.34)
Preterm birth, *n* (%)	2114 (4.94)	2003 (5.04)	111 (3.66)
Parity, *n* (%) *			
0 (no older siblings)	20,926 (48.88)	19,732 (49.61)	1194 (39.33)
1	15,630 (36.51)	14,373 (36.13)	1257 (41.40)
≥2	6256 (14.61)	5671 (14.26)	585 (19.27)
Family numbers, *n* (%) *			
1	308 (0.72)	290 (0.73)	18 (0.59)
2	11,564 (27.01)	10,958 (27.55)	606 (19.96)
3~5	27,542 (64.33)	25,486 (64.07)	2056 (67.72)
≥6	3396 (7.93)	3040 (7.64)	356 (11.73)
Infant feeding practice, *n* (%) †			
Formula feeding without colostrum	663 (1.56)	623 (1.58)	40 (1.33)
Partial breastfeeding	32,644 (76.83)	30,572 (77.42)	2072 (69.11)
Exclusive breastfeeding to 6–7 months of age	9181 (21.61)	8295 (21.01)	886 (29.55)
Daycare attendance, *n* (%) ‡			
No	26,049 (77.20)	17,577 (78.29)	4811 (75.05)
Yes	7595 (22.51)	8472 (21.43)	2784 (24.66)
Residential area, *n* (%) *			
Wards	9388 (21.93)	8820 (22.17)	568 (18.71)
Cities	25,205 (58.87)	23,410 (58.85)	1795 (59.12)
Towns or villages	8219 (19.20)	7546 (18.97)	673 (22.17)
Characteristics of parents			
Mean maternal age at delivery, years (SD) *	30.13 (4.42)	30.13 (4.41)	30.15 (4.64)
Mean paternal age at delivery, years (SD) *	32.30 (5.53)	32.29 (5.51)	32.41 (5.84)
Maternal smoking status, *n* (%) †			
Non-smoker	35,882 (84.24)	33,425 (84.44)	2457 (81.60)
Smoker	6711 (15.76)	6157 (15.56)	554 (18.40)
Maternal educational attainment, *n* (%) ‡			
University or higher	5849 (14.12)	5538 (14.38)	311 (10.67)
Junior college	17,305 (41.76)	16,197 (42.05)	1108 (38.01)
Less than or equal to high school	18,284 (44.12)	16,788 (43.58)	1496 (51.32)
Paternal educational attainment, *n* (%) ‡			
University or higher	15,134 (36.89)	14,239 (37.33)	895 (31.07)
Junior college	6453 (15.73)	6020 (15.78)	433 (15.03)
Less than or equal to high school	19,435 (47.38)	17,882 (46.88)	1553 (53.90)

* Obtained from the birth record. † Obtained from the first survey (at the age of 6 months). ‡ Obtained from the third survey (at the age of 2.5 years).

**Table 2:** Caries Status and Influenza from the Third, Sixth and Tenth Surveys.

		Crude	Model 1	Model 2
	Ncase/N(%)	RR(95% CI)	† aRR(95% CI)	‡ aRR(95% CI)
Between the ages of 1.5 and 2.5 years				
caries (−)	5433/39,776(13.66)	1 (reference)	1 (reference)	1 (reference)
caries (+)	488/3036(16.07)	1.18(1.08–1.28)	1.15 (1.06–1.25)	1.15 (1.05–1.25)
Between the ages of 4.5 and 5.5 years				
caries (−)	3683/24,497(15.03)	1 (reference)	1 (reference)	1 (reference)
caries (+)	2231/14,043(15.89)	1.06(1.01–1.11)	1.07 (1.02–1.12)	1.06 (1.01–1.11)
Between the ages of 9 and 10 years				
caries (−)	4035/22,715(17.76)	1 (reference)	1 (reference)	1 (reference)
caries (+)	2446/11,409(21.44)	1.21(1.15–1.26)	1.21(1.16–1.27)	1.22 (1.17–1.28)

CI, confidence interval; RR, risk ratio. † Adjusted for child factors (sex, singleton or not, gestational age, infant nutrition, parity, family number, daycare attendance at the age of 18 months and birth and residential places). ‡ Adjusted for child factors (sex, singleton or not, gestational age, infant nutrition, parity, family number, daycare attendance at the age of 18 months and birth and residential places) as well as parental factors (maternal age at delivery, paternal age at delivery, maternal smoking status, maternal educational attainment, and paternal educational attainment).

**Table 3:** Caries Status and Influenza between the Ages of 9 and 10 Years: Subgroup analysis by Previous Caries Status.

	Ncase/*N*(%)	RR(95% CI)	† aRR(95% CI)	‡ aRR(95% CI)
Previous caries (−)				
caries (−) between the ages of 9 and 10	1495/8322(17.96)	1 (reference)	1 (reference)	1 (reference)
caries (+) between the ages of 9 and 10	199/895(22.23)	1.19(1.12–1.27)	1.24(1.09–1.41)	1.23(1.08–1.41)
Previous caries (+)				
caries (−) between the ages of 9 and 10	2343/13321(17.59)	0.98(0.92–1.04)	0.99(0.94–1.06)	1.01(0.95–1.07)
caries (+) between the ages of 9 and 10	2222/10367(21.43)	1.24(1.09–1.41)	1.22(1.15–1.29)	1.23(1.16–1.30)

CI, confidence interval; RR, risk ratio. † Adjusted for child factors (sex, singleton or not, gestational age, infant nutrition, parity, family member number, daycare attendance at the age of 18 months and birth and residential places). ‡ Adjusted for child factors (sex, singleton or not, gestational age, infant nutrition, parity, family member number, daycare attendance at the age of 18 months and birth and residential places) as well as parental factors (maternal age at delivery, paternal age at delivery, maternal smoking status, maternal educational attainment, and paternal educational attainment).

**Table 4:** Caries Status and Influenza between the Ages of 9 and 10 Years Stratified by House Income.

	House Income (yen)					
	Low Income		Middle Income		High Income	
	Ncase/*N*(%)	aRR(95% CI)	Ncase/*N*(%)	aRR(95% CI)	Ncase/*N*(%)	aRR(95% CI)
caries (−) between the ages of 9 and 10	376/2354 (15.97)	1 (reference)	2461/13,421 (18.34)	1 (reference)	489/2733 (17.89)	1 (reference)
caries (+) between the ages of 9 and 10	542/2438 (22.23)	1.34 (1.21–1.48)	995/4609 (21.59)	1.19 (1.11–1.27)	462/2216 (20.85)	1.16 (1.04–1.28)

Low income (<4,780,000), Middle income (4,780,000~8,200,000), High income (>=8,200,000). CI, confidence interval; RR, risk ratio. Adjusted for child factors (sex, singleton or not, gestational age, infant nutrition, parity, family member number, daycare attendance at the age of 18 months and birth and residential places) as well as parental factors (maternal age at delivery, paternal age at delivery, maternal smoking status, maternal educational attainment, and paternal educational attainment).

## Data Availability

The data that support the findings of this study are available from The Japanese Ministry of Health, Labour and Welfare, but restrictions apply to the availability of these data, which were used under license for the current study, and so are not publicly available. Data are, however, available from the authors upon reasonable request and with permission of The Japanese Ministry of Health, Labour and Welfare.
